# Smart Speaker Recommendations: Impact of Gender Congruence and Amount of Information on Users' Engagement and Choice

**DOI:** 10.3389/fpsyg.2021.659994

**Published:** 2021-04-08

**Authors:** Jaime Romero, Daniel Ruiz-Equihua, Sandra Maria Correia Loureiro, Luis V. Casaló

**Affiliations:** ^1^Departamento de Financiación e investigación Comercial: UDI de Marketing, Autonomous University of Madrid, Madrid, Spain; ^2^Departamento de Marketing, Operações e Gestão Geral, Instituto Universitário de Lisboa (ISCTE) Business School, University Institute of Lisbon, Lisbon, Portugal; ^3^Departamento de Dirección de Marketing e Investigación de Mercados Área de Comercialización e Investigación de Mercados, University of Zaragoza, Zaragoza, Spain

**Keywords:** smart speakers, visiting intentions, customer attitudes, customer engagement, gender congruence, amount of information

## Abstract

The relevance of smart speakers is steadily increasing, allowing users perform several daily tasks. From a commercial perspective, smart speakers also provide recommendations of products and services that may influence the consumer decision-making process. However, previous studies have mainly focused on the adoption of smart speakers, but there is a lack of proper guidelines that help design the way these devices should offer their consumption recommendations. Based on a stimulus-organism-response approach, we analyze how two features of smart speakers' recommendations (the gender congruence between the customer and the speaker, and the length of the message) influence on the effectiveness of such recommendations (i.e., visiting intentions) through its impact on user engagement and attitude. Data was collected from a sample of undergrad students in Spain using an experiment design that focused on a restaurant recommendation, and analyzed using partial least squares. On the one hand, our results suggests that gender congruence generates user engagement with the smart speaker. On the other hand, message length is positively related to attitudes towards the restaurant, at a declining rate. In addition, while better attitudes lead to higher visiting intentions, the influence of engagement on visiting intentions is partially mediated via attitudes. Thus, our findings contribute to understand the antecedents of users' engagement with smart speakers, as well as its impact on the customers' willingness to follow smart speakers' recommendations, constituting a base to analyze the impact of artificial intelligence solutions aimed to smooth the transitions of a customer through the stages of purchase process.

## Introduction

Smart speakers help individuals to perform a range of simple tasks in everyday life, such as reporting the weather forecast, switching off the light, or playing music. They can also recommend stores, products, services, etc., fitting customer requirements. These devices based on artificial intelligence have the ability to interact and converse with humans (e.g., Belanche et al., 2020). Specifically, smart speakers offer several applications in the hospitality industry, such ordering room service (e.g., Marriot use of Echo devices in select hotel rooms), booking a rental car or hotel, tracking flight prices and status or suggesting nearby restaurants (Hornick and Santhanam, 2018). With a 20% expected annual growth rate and projected sales of more than 500 million units worldwide in 2024 (Wadhwani and Gankar, 2018), the potential influence of smart speakers recommendations is huge. However, customers cannot process voice-based information as efficiently as visual or even text-based information, mostly because of a lack of accuracy and user-system interaction (Lee and Pan, 2010), so smart speakers need to offer engaging recommendations that generate favorable attitudes toward the recommended product or service as well as purchase or visiting intentions.

Past research has not analyzed yet how the engagement process evolves between humans and smart speakers; particularly, how such engagement influences the customer attitude and intention toward the recommendation of a store, brand, or firm (Loureiro et al., in press). Smart speaker developers lack proper guidelines helping them design the way these devices should offer their recommendations to customers. This research aims to fill this gap. Specifically, we analyze how two features of smart speaker recommendations (namely gender congruence between customer and speaker, and amount of information in the message) influence the effectiveness of such recommendations.

**Figure 1:**
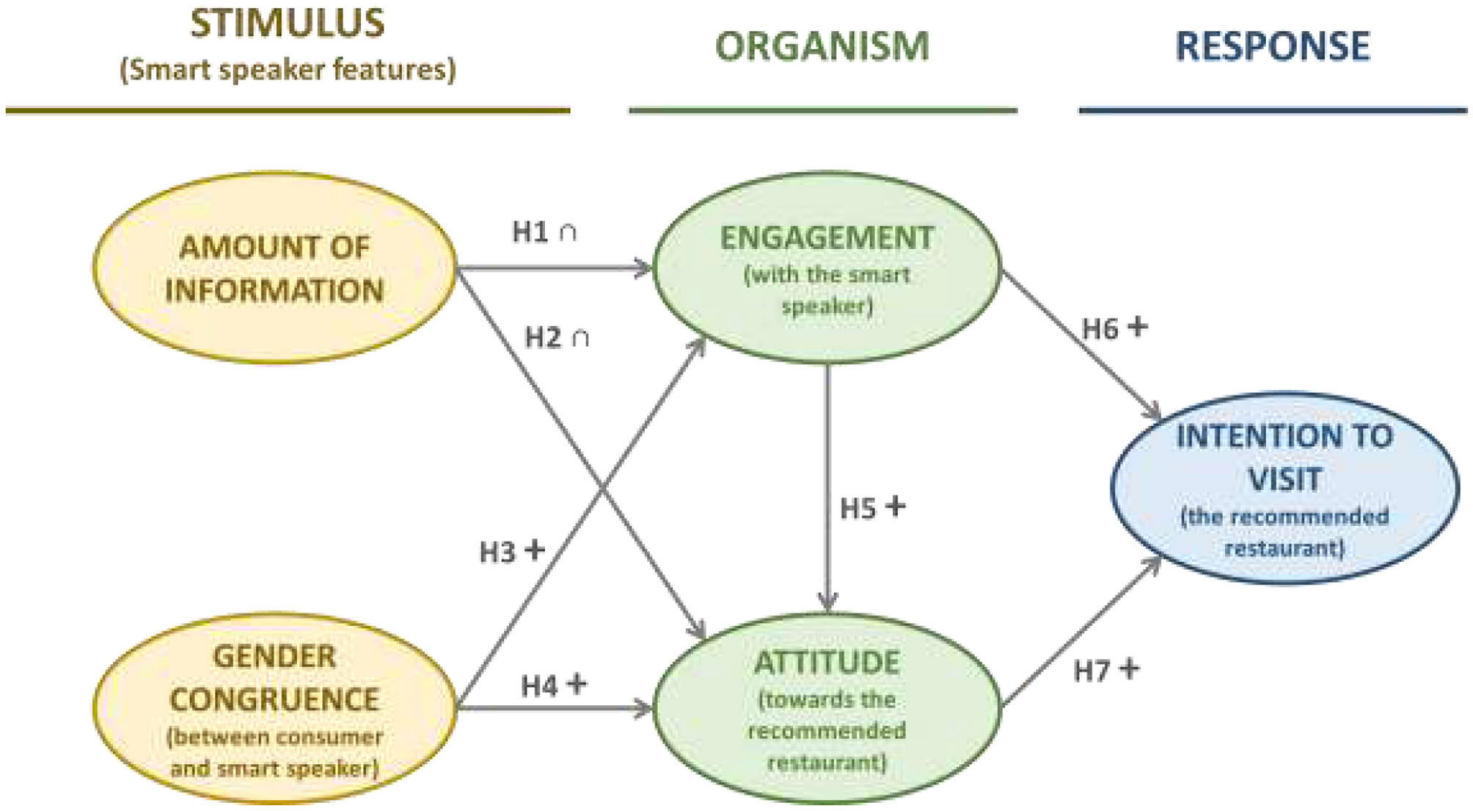
Theoretical framework.

To do so, we follow the S(Stimuli)-O(Organism)-R(response) framework, which we apply to restaurant recommendations. The *stimuli* (attributes or features) comprise drivers affecting users' cognitive and emotional states (*organism*), whose responses may be either approach or avoidance (*responses*) (Roschk et al., 2017). For this research, the stimulus includes the smart speaker recommendation features. The organism comprises user engagement with the smart speaker and attitude toward the recommended restaurant. The response is the approach, that is, the visiting intention (Figure 1).

Regarding the recommendation, we consider two important features: the amount of information provided by the smart speaker and the gender congruence between the smart speaker and the customer. The amount of information is defined as how much information is provided in the smart speaker recommendation message. This aspect is very relevant in information processing (Bagozzi et al., 2016). Gender congruence occurs when the gender of the information source (the smart speaker in this case) is the same as that of the customer. The influence of an information source may differ depending on the gender of both the information source and the receiver of the message (e.g., Casaló and Escario, 2016).

Customer engagement with a smart speaker captures customer interactions with a firm through this device (Bilro and Loureiro, 2020). Customer engagement as “a psychological state that occurs by virtue of interactive, co-creative customer experiences with a focal agent object” (Brodie et al., 2011), such as a smart speaker. Hollebeek et al. (2014) note that, during interaction between the customer and the firm – represented here by the smart speaker – customer engagement embraces cognitive, emotional, and behavioral activities (paying attention to the interaction, having feelings toward the firm through the smart speaker, and actually using the device). Engaged customers tend to feel satisfaction (Brodie et al., 2013; Fehrer et al., 2018), become loyal (Hollebeek, 2011; Dwivedi, 2015), and say positive things about the firm (Vivek et al., 2012; Hollebeek et al., 2014; McLean and Wilson, 2019).

Customer attitudes are one of the main outcomes generated by product/service recommendations (Casaló et al., 2015a; Ruiz-Equihua et al., 2020). Attitudes capture the assessments that customers make regarding a behavior (Wu and Chen, 2005) – visiting a recommended restaurant in this case. Customer attitudes arising from a recommendation determine their intention to follow such recommendation. According to the Theory of Planned Behavior (Ajzen, 1991), behavioral intentions reflect a person's willingness to perform a specific behavior. Therefore, the intention to visit the recommended restaurant may be a good indicator of the actual customer behavior as behavioral intentions imply that customers will likely behave in a specific way (McKnight et al., 2002).

Consistent with the S-O-R framework, we first propose that amount of information and gender congruence influence both customer engagement with the smart speaker and attitudes toward the recommended restaurant.

The amount of information provided by the smart speaker may be perceived as a sign of its interactivity, which may cause greater engagement (Sundar, 2007; Blasco-Arcas et al., 2013; Liao et al., 2019). In addition, customers may pay more cognitive attention to messages with a higher amount of information, resulting in a greater engagement with the device. Longer messages recommending products are considered more useful (Yang et al., 2017) and generate more sales (Chevalier and Mayzlin, 2006) than shorter ones. Therefore, we expect that the more information reinforcing a purchase decision, the more favorable the attitude toward the recommended product will be. We expect a similar effect on customer engagement, as the information delivered by the smart speaker aims to develop attention and affection, and trigger an interaction with the smart speaker (Hollebeek et al., 2014, 2019). However, the elaborateness of the information generated by firms does not produce a positive effect on customers if it is too short or too long (Hernández-Ortega et al., 2020). Therefore, we expect that this will occur at a declining rate, even possibly reaching a saturation point (Kim et al., 2001).

H1: Amount of information is positively associated with customer engagement with the smart speaker at a declining rate.
H2: Amount of information is positively associated with customer attitudes toward the recommended restaurant at a declining rate.

Previous research indicates that the influence of an information source depends on its similarity with the message receiver (Casaló and Escario, 2016). Specifically, customers perceiving a higher similarity with the information source (i.e., the smart speaker) tend to consider that the source's opinions are congruent with their own personal values (Casaló et al., 2011), being influenced more easily by such opinions. Particularly, gender congruence between the message sender and receiver generates more positive perceptions about the sender (Jordán, 2015); for example, a higher trust, leading customers to engage with the congruent source. Gender congruence also serves to internalize the transmitted message (Casaló et al., 2013), provoking a more favorable attitude toward it. Thus, we expect that:

H3: Gender congruence is positively associated with customer engagement with the smart speaker.
H4: Gender congruence is positively associated with customer attitude toward the recommended restaurant.

Customer engagement and attitude toward the recommended restaurant represent the organism of the S-O-R framework. Customers engaged with a smart speaker will think a lot about the information provided by the smart speaker, will spend a lot of time in their interactive activity, and will be happy and proud to use the smart speaker (Hollebeek et al., 2014). Since engaged individuals are more likely to develop more favorable attitudes (Vivek et al., 2012), we argue that these engaged customers are expected to have a favorable assessment (Wu and Chen, 2005) toward the recommended restaurant, leading to the following hypothesis:

H5: Customer engagement with the smart speaker is positively associated with customer attitude toward the recommended restaurant.

As the S-O-R framework explains, an organism creates a response. Traditionally, response is associated with behavioral intentions (Roschk et al., 2017). Prior studies have considered behavior intentions (representing the willingness to visit, re-purchase, or recommend to others) as outcomes of engagement (e.g., Hollebeek, 2011; Vivek et al., 2012) and of favorable attitudes (Casaló et al., 2015a; Ruiz-Equihua et al., 2020). While engagement implies a strong psychological connection that may lead to positive outcomes such as behavioral intentions, attitude plays a relevant role in forming consumer preferences (Moriuchi, 2019). In this vein, we propose that customer engagement with the smart speaker and attitude toward the recommended restaurant influence customer visiting intentions:

H6: Customer engagement with the smart speaker is positively associated with the intention to visit the recommended restaurant.
H7: Customer attitudes toward the recommended restaurant are positively associated with the intention to visit the recommended restaurant.

For the sake of completeness, we include two control variables in our model: customer gender and expertise with smart speakers: women and men process information in a different way (Venkatesh and Morris, 2000); experience with the smart speaker makes customers more familiar with and more knowledgeable about the smart speaker (Sun and Zhang, 2006). Therefore, gender and experience may influence customers' beliefs, evaluations, and intentions.

## Methods

We tested our hypotheses about the effect of smart speaker recommendations in customer behaviors using a 3 (short, medium and large amount of information) × 2 (male smart speaker voice vs. female smart speaker voice) experimental design. Respondents include undergraduate “Business Administration” students from Universidad Autónoma de Madrid, second and fourth year, who participated in the study during March 2020 (before COVID-19 mobility restrictions), in exchange for course credits (*n* = 270; Table 1 contains sample demographics). We presented them a situation where they got a new smart speaker; after installing it and familiarizing themselves with its functions, they asked for a recommendation for dinner at a downtown restaurant. First, we manipulated the amount of information through three restaurant recommendations of low, medium and high duration. The shortest message included three basic attributes to reach the restaurant (name, address, and schedule), lasting 4 s. The longest message contained nine attributes (adding rating, price, cuisine type, and extra features, namely wi-fi, credit card accepted, and accessibility), lasting 20 s and providing a high amount of information without overloading respondents (Lee and Lee, 2004). Between both anchors, the intermediate message included six attributes (no extra features), lasting 12 s. Second, we manipulated gender congruence by modifying the voice of the smart speaker (female vs. male). We randomly assigned respondents to each condition and asked them to answer a survey containing questions about their engagement with smart speakers, attitudes toward the restaurant, and visiting intentions. We also included realism and manipulation check questions. We guaranteed participant anonymity and induced a psychological separation between our variables by including questions not related to the research goals to avoid common method bias problems (Podsakoff et al., 2003). The questionnaire was implemented in Qualtrics and self-administered by participants.

**Table 1:** Sample profile.

	**Male**	**Female**	**Total**
<20	18.89	21.48	40.37
20–24	28.89	28.15	57.04
25–34	0.74	0.74	1.48
35–44	0.37	0.00	0.37
45–54	0.74	0.00	0.74
Total	49.63	50.37	100

*All figures are in percentages*.

### Measures

We employed scales from prior research in our study for engagement, customer attitude, visiting intentions, and experience with smart speakers (Wu and Chen, 2005; Hollebeek et al., 2014; Casaló et al., 2015b; Matzler et al., 2016). We adapted these scales to our research context, measured amount of information and customer gender congruence using single-item scales. Respondents also reported their gender.

We measured all our variables as first-order construct, except customer engagement with the smart speaker. Consistent with its three-dimensional conceptualization, which includes cognitive processing, affection and activation (Hollebeek et al., 2014) we measured it as a type II reflective-formative second-order construct (Ringle et al., 2012). Respondents assessed the first-order constructs of our research using seven-point Likert-type scales, except for gender (male/female/prefer not to answer).

## Results

We estimate our model using partial least squares (PLS). First, PLS is an appropriate method to develop theories in exploratory research, as in our case. Second, PLS can properly handle sample size such as the one in our study. Third, PLS can properly estimate type II constructs, such as customer engagement with the smart speaker in our research. Given that customer engagement with the smart speaker is an endogenous construct in our research, we estimate our model using a two-stage approach (Ringle et al., 2012).

### Manipulation Check and Common Method Bias Assessment

We measured scenarios' realism and credibility using the following items taken from Bagozzi et al. (2016): “the scenario is realistic,” “the scenario is credible,” “how likely is it that the smart speaker would give you advice like the one that you hear here?” (all items were measured using a 7-point Likert scale). The items provided a reliable measure of realism and credibility (Cronbach's α = 0.71), which were computed as the average of the three items. The results confirmed the suitability of the scenarios. The mean of the measure was 5.45 (standard deviation = 1.11, hereafter, SD), significantly >4 – the central point of the scale (*t* = 80.66, *p* < 0.01).

Additionally, we tested the manipulation of the amount of information using one item: “the quantity of information provided by the smart speaker is …,” with answers ranging from 1, “insufficient” to 7, “excessive.” The scenarios obtained a mean of 4.25 (SD = 0.98), 4.91 (SD = 0.83), and 5.24 (SD = 0.94) for the low, medium, and high information conditions, respectively. The respondents perceived the information scenarios as different (t_low−medium_ = −4.85, *p* < 0.01; t_medium−high_ = −2.48, *p* < 0.05; *t*_low−high_ = −6.94, *p* < 0.01), hence confirming a successful manipulation.

We also assessed the manipulation of gender congruence. The item used was “smart speaker gender is …” with answers ranging from 1, “different from mine” to 7, “equal to mine.” Respondents reported a higher congruence when exposed to a voice corresponding to their gender than when not exposed (M_males−different_ = 2.43, SD = 1.90, vs. M_males−same_ = 4.73, SD = 2.47; M_females−different_ = 1.55, SD = 1.28, vs. M_females−same_ = 5.18, SD = 2.37), being such differences significant (t_males_ = 5.97, *p* < 0.01; *t*_females_ = 10.92, *p* < 0.01). Therefore, our respondents successfully perceived our gender congruence manipulation.

Finally, we assessed whether common method bias is a problem in our study by evaluating variance inflation factors (Kock and Lynn, 2012). They all range between 1.01 and 2.24, below the recommended 3.3 threshold.

### Measurement Model

We first evaluated the reliability and convergent validity of the first order construct in our model (Table 2). Cronbach's alpha ranges between 0.79 and 0.94, above the 0.7 ordinary threshold value (Nunnally, 1978). Composite reliability oscillates between 0.88 and 0.95. The loadings of constructs indicators are all above 0.7, supporting then indicator reliability. The average variance extracted (AVE) varies between 0.69 and 0.84, hence higher than the cut-off value of 0.5 suggested by Fornell and Larcker (1981).

**Table 2:** Measurement items.

**Item**	**Mean**	**SD**	**Excess Kurtosis**	**Skewness**
Amount of information (α = NA; CR = NA; AVE = NA)				
The amount of information given by the smart speaker is…	4.80	0.06	1.19	−0.45
Customer gender congruence (α = NA; CR = NA; AVE = NA).				
The smart speaker gender is…	3.56	0.15	−1.16	0.31
Engagement Cognitive (α = 0.79; CR = 0.88; AVE = 0.70) (Hollebeek et al., 2014) The interaction with this smart speaker…				
Makes me think about a smart speaker	3.91	0.09	−0.41	−0.12
Generates my learning about and interest in smart speakers	3.88	0.09	−0.95	−0.04
Makes me think about smart speakers	4.46	0.10	−0.85	−0.34
Affective (α = 0.90; CR = 0.94; AVE = 0.84) (Hollebeek et al., 2014) The interaction with this smart speaker…				
Makes me feel good	3.33	0.08	−0.65	0.15
Makes me feel happy	2.90	0.08	−0.55	0.36
Makes me feel proud of using it	3.07	0.09	−0.81	0.33
Activation (α = 0.85; CR = 0.91; AVE = 0.77) (Hollebeek et al., 2014)				
I would spend a lot of time using smart speakers compared to other information sources	3.27	0.09	−0.56	0.39
I would use a smart speaker	4.69	0.10	−0.57	−0.54
Smart speakers would be one of the devices that I would use	4.39	0.10	−0.75	−0.29
Customer attitude (α = 0.89; CR = 0.92; AVE = 0.69) (Wu and Chen, 2005; Casaló et al., 2015b)				
I have a positive opinion about this restaurant	4.58	0.08	0.27	−0.46
I think that visiting this restaurant is a good idea	4.99	0.07	0.33	−0.51
I think that visiting this restaurant is a wise idea	4.49	0.08	0.06	−0.24
I think that visiting this restaurant is an appropriate idea	4.69	0.07	0.07	−0.36
Visiting this restaurant would be a pleasant experience	4.96	0.07	0.05	−0.46
Customer visiting intentions (α = 0.94; CR = 0.95; AVE = 0.80) (Casaló et al., 2015b; Matzler et al., 2016)				
I can imagine visiting this restaurant	4.61	0.09	−0.28	−0.55
I think that I could visit this restaurant	4.93	0.09	0.11	−0.73
I intended to visit this restaurant soon	4.57	0.10	−0.37	−0.60
If I needed it, I would likely visit this restaurant	4.89	0.09	0.01	−0.69
I intend to search for this restaurant to visit soon	4.01	0.09	−0.48	−0.14
Experience with smart speakers (α = 0.91; CR = 0.94; AVE = 0.84).				
I am familiarized with using voice assistants…	4.46	0.10	−0.92	−0.24
I have experience using voice assistants…	4.14	0.11	−1.11	0.02
I am an experimented user of voice assistants…	3.21	0.10	−0.76	0.44

*All items are measured with a 7-point Likert scale anchoring strongly disagree (1) and strongly agree (7); except for amount of information, it included a single item 7-point scale ranging from (1) insufficient to (7) excessive; and customer gender congruence, it included a single item 7-point scale ranging from (1) different from mine to (7) the same as mine. α = Cronbach's alpha, CR = composite reliability, AVE = average variance extracted, SD = standard deviation*.

We next evaluated the discriminant validity of our variables using three criteria. We first evaluated whether the loadings of each indicator are higher for its assigned variable than for other variables. Second, we apply the Fornell and Larcker (1981) criterion. We checked whether the square root of the latent variables' AVE is higher that their correlations with other variables (Table 3). Finally, we computed the heterotrait-monotrait ratio (HTMT) of the correlations and checked whether they are lower than 0.85 to support discriminant validity (Clark and Watson, 1995; Kline, 2011). The cross-loadings of our items, the Fornell and Larcker's (1981) criterion, and the HTMT values support the discriminant validity of our variables.

**Table 3:** Discriminant validity analysis.

**Fornell and Larcker**	**(1)**	**(2)**	**(3)**	**(4)**	**(5)**	**(6)**	**(7)**	**(8)**	**(9)**
Amount of information (1)	1.00								
Congruence (2)	−0.03	1.00							
Engagement - Cognitive (3)	0.00	0.02	0.84						
Engagement - Activation (4)	0.05	0.13	0.31	0.88					
Engagement - Affective (5)	0.07	0.07	0.56	0.39	0.92				
Attitude (6)	0.29	0.00	0.28	0.21	0.33	0.83			
Intention (7)	0.26	0.01	0.24	0.25	0.21	0.71	0.90		
Gender (8)	−0.12	0.03	−0.10	−0.02	−0.09	0.03	−0.08	1.00	
Expertise (9)	−0.03	0.03	0.20	0.35	0.22	0.11	0.08	0.03	0.92
**HTMT:**									
Amount of information (1)									
Congruence (2)	0.03								
Engagement - Cognitive (3)	0.05	0.07							
Engagement - Activation (4)	0.05	0.14	0.37						
Engagement - Affective (5)	0.07	0.08	0.66	0.45					
Attitude (6)	0.31	0.04	0.33	0.24	0.37				
Intention (7)	0.27	0.04	0.28	0.28	0.23	0.77			
Gender (8)	0.12	0.03	0.11	0.03	0.10	0.05	0.08		
Expertise (9)	0.03	0.03	0.24	0.40	0.24	0.12	0.09	0.06	

*Numbers on the diagonal for the Fornell and Larcker crtierion show the square root of the average variance extracted; numbers below the diagonal represent construct correlations*.

After evaluating the measurement model of our first-order constructs, we re-estimated the model incorporating the latent scores of cognitive, affective, and activation as customer engagement indicators. Subsequently, we assessed the measurement model of customer engagement with the smart speaker through the significance of its indicators (calculated employing a non-parametric bootstrapping procedure with 10,000 subsamples, no sign change). They are all significant at a 95% level. Their variance inflation factors vary between 1.16 and 1.50, ensuring the required lack of collinearity in the measurement of customer engagement (Diamantopoulos and Winklhofer, 2001).

### Structural Model

Regarding our structural model, we first assessed the global fit of the model through its standardized root mean residual (SRMR). The SRMR of our model is 0.05, below 0.08, thus indicating an adequate global fit (Hu and Bentler, 1998). Subsequently, we assessed how our model explains each endogenous variables through adjusted-R^2^. These are for 0.13 engagement,0.19 for attitude, and 0.51 for visiting intentions, showing a medium fit for visiting intentions, and small fit for attitude and engagement (Chin, 1998). Next, the Q^2^ values for customer engagement (0.05), attitude (0.13), and visiting intentions (0.41) indicate medium predictive relevance for engagement and attitude, and high for visiting intentions (Hair et al., 2017).

Finally, we calculated the significance of our path estimates. We employed a non-parametric bias corrected and accelerated bootstrapping procedure with 10,000 sub-samples, no sign change. We showed our path estimates, *t* estimates and *p*-values in Table 4.

**Table 4:** Structural relationships.

**Structural relationship**	**ß**	**SD**	***t***	***p*-value**	**Hypothesis**
Amount of information^2^ -> Engagement	−0.00	0.04	0.21	0.83	H1rejected
Amount of information -> Engagement	0.06	0.06	1.07	0.28	
Amount of information ^2^ -> Attitude	−0.09	0.04	2.23	0.02	H2 supported
Amount of information -> Attitude	0.27	0.07	4.16	0.00	
Congruence -> Engagement	0.09	0.06	1.65	0.09	H3 supported (90%)
Congruence -> Attitude	−0.02	0.05	0.53	0.59	H4 rejected
Engagement -> Attitude	0.31	0.06	4.96	0.00	H5 supported
Engagement -> Intention	0.09	0.06	1.85	0.06	H6 supported (90%)
Attitude -> Intention	0.68	0.04	18.79	0.00	H7supported
Expertise -> Attitude	0.02	0.06	0.36	0.71	
Expertise -> Engagement	0.35	0.06	5.71	0.00	
Expertise -> Intention	−0.02	0.04	0.48	0.63	
Gender -> Attitude	0.10	0.05	1.85	0.06	
Gender -> Engagement	−0.08	0.06	1.46	0.14	
Gender -> Intention	−0.09	0.04	2.15	0.03	

H1 proposes that the amount of information has a positive effect on customer engagement, at a declining rate. Neither the linear path estimate (0.06; *p*-value: 0.28) nor the quadratic path estimate (−0.00; *p*-value: 0.83) that capture the effect of amount of information on engagement are significant. Therefore, we reject H1. The length of the recommendation provided by the smart speaker does not influence the engagement of the customer with this device.

H2 posits that the amount of information has a positive effect on customer attitude, at a declining rate. Both the linear path estimate (0.27; *p*-value: <0.01) and the quadratic path estimate (−0.09; *p*-value: 0.02) that capture the effect of amount of information on customer attitude are significant. Hence, our results support H2. Particularly, our results indicate that the more information provided to the customer, the higher the attitude toward the recommended restaurant. The negative sign of the quadratic effect captures the declining impact of the amount of information; the amount of information provokes better attitudes, but this effect is lower as the amount of information grows.

H3 suggests that gender congruence positively influences customer engagement with the smart speaker. Our results support H3 only at a 90% level (0.09; *p*-value: 0.09). Individuals tend to engage more with smart speakers when they consider that the smart speaker has the same gender as them.

H4 evaluates whether gender congruence positively influences attitude toward the recommended restaurant. According to our results, this influence does not exist (−0.02; *p*-value: 0.59). We reject H4. The gender of the smart speaker voice does not influence attitude toward the recommended restaurant.

H5 indicates that customer engagement with the smart speaker is positively associated with attitude toward the recommended restaurant. Our results support H5 (0.31; *p*-value: <0.01). The more engaged with the smart speaker the customer is, the better attitude the smart speaker recommendation generates.

According to H6, customer engagement with the smart speaker increases the intention to visit the recommended restaurant. Our results support H6 at a 90% level (0.09; *p*-value: 0.06). Customer engagement with the smart speaker has a direct influence on intention to follow the recommendation made by the device.

Finally, H7 proposes that attitudes toward the restaurant are positively associated with the intention to visit the recommended restaurant. Our results support H7 (0.68; *p*-value: <0.01). The higher the attitudes toward the recommended restaurant, the higher the intention to visit it.

Together, H5 and H7 suggest that engagement might also indirectly influence intention, mediated by attitudes. This indirect effect is indeed significant (0.21; *p*-value: <0.01), according to our bootstrapping procedure. Thus, the total effect of engagement on intention is also significant (0.30; *p*-value: <0.01).

## Discussion and Implications

Following the well-established S-O-R model (e.g., Mehrabian and Russell, 1974; Donovan and Rossiter, 1982), this research studies how smart speaker features, namely the amount of information and gender congruence with the consumer, influence intention to follow the recommendation due to their impact on customer engagement with the smart speaker and on attitude toward the recommended restaurant. Aligned with what previous studies suggest (Sundar, 2007; Blasco-Arcas et al., 2013; Liao et al., 2019), our results indicate that gender congruence generates user engagement with the smart speaker. Amount of information is positively related with attitude toward the restaurant, at a declining rate, that is, this impact is lower as the amount of information increases. Better attitude leads to higher visiting intention. In contrast, the influence of engagement on visiting intention is mainly indirect, via attitude. These findings have important theoretical and practical implications.

From a theoretical perspective, this research contributes to previous literature in two ways. First, while amount of information seems to be related to attitude toward the recommended product (with a declining impact, similar to previous research findings, e.g., Hernández-Ortega et al., 2020), gender congruence is more related to engagement with the smart speaker. The reason behind this finding may be based on the key elements of communication (Chandler, 1994). The amount of information represents a characteristic of the message transmitted. In contrast, gender congruence depends on the characteristics of the sender and the receiver of the message. Therefore, the amount of information affects more a variable related to the content of the message (attitude toward the recommended product), and gender congruence affects more a variable related to the sender of the message (engagement with the smart speaker). The influence of congruence could be explained by the similarity between the customer and the smart speaker, which may derive in a greater consumer identification with the sender of the message (e.g., Schouten et al., 2020).

Second, engagement with the smart speaker may serve to develop affective feelings –and even behavioral intentions– toward the products/services recommended by the smart speaker. Specifically, the influence of engagement on visiting intentions is partially mediated by attitude. This result complements previous literature, which has mostly analyzed customer engagement with brands within technological environments (e.g., Hollebeek et al., 2019), suggesting that this engagement may have positive consequences for both the brand and the technology (e.g., McLean and Wilson, 2019; Bilro and Loureiro, 2020).

From a managerial perspective, smart speaker developers should be aware that they must ensure gender congruence with users. Users tend to be more engaged – meaning that they spend more time using the smart speaker, are happier and prouder, and gain interest regarding smart speakers – when their own gender fits the perceived voice of the smart speaker. Currently, the most popular smart speakers (e.g., Alexa, Siri, Cortana) do not allow their users selecting assistants' gender. Manufacturers should incorporate this feature. Additionally, the smart speaker should be able to capture the sensibility of users to give the amount of information to captivate users, rather than bore or irritate them. The intention to visit the restaurant will only be created in the user's mind if the smart speaker can develop a favorable attitude toward the restaurant, that is, when users think that visiting the restaurant is a good idea. Therefore, the learning process of the smart speaker should be such that it provides an adequate amount of information, and uses a voice that is congruent to the users to develop a sense of interaction and a favorable evaluation of the service provided.

Our study is not exempt from limitations, which constitute opportunities for further research. First, we have employed a convenience sample (i.e., students) in our study. Consequently, our findings circumscribe to our sample and cannot be generalized. Hence, further research could replicate our model employing representative samples. Similarly, we have just considered one recommended product/service, a restaurant. Generalizing our results would require testing our model with a variety of different products and services (e.g., high vs. low involvement, familiar vs. unfamiliar brands, etc.). The recommendation features could be also expanded by further research, thus providing a better comprehension of how message features influence individuals. This is especially relevant as the percentage of variance explained for attitudes and engagement, although adequate for studies that address novel topics such as ours, is not very high. For example, language style (e.g., figurative vs. literal) might influence the way in which consumers reacts to smart speaker recommendations (as in the case of online reviews; Wu et al., 2017). Additionally, we suggest exploring the concept of “coolness” (Warren et al., 2019) and whether or not a smart voice regarded as “cool” by users – due to the tone of voice and the kind of information provided – will generate more engagement and consequently reinforce the intention to visit a restaurant or other place. Further research could also focus on additional consumer characteristics, such as technology readiness (Belanche et al., 2020), to identify the potential drivers of engagement with smart speakers. Finally, we adopt a static perspective in our research. Adopting a dynamic perspective to study this phenomenon would allow understanding whether the effects of message features remain stable across time.

## Data Availability Statement

The datasets presented in this article are not readily available because they were gathered under no-distribution assurance. Requests to access the datasets should be directed to Daniel Ruiz-Equihua, daniel.ruize@uam.es.

## Ethics Statement

Ethical review and approval was not required for the study on human participants in accordance with the local legislation and institutional requirements. Written informed consent for participation was not required for this study in accordance with the national legislation and the institutional requirements.

## Author Contributions

All authors contributed to the study conception and design. Material preparation, data collection and analysis were prepared by LC, DR-E, and JR. The first draft of the manuscript was written by JR, and subsequently extended and refined by LC, SL, and DR-E. All the authors read and approved the final version of the manuscript.

## Conflict of Interest

The authors declare that the research was conducted in the absence of any commercial or financial relationships that could be construed as a potential conflict of interest.
